# Aerobic exercise training protects against endothelial dysfunction by increasing nitric oxide and hydrogen peroxide production in LDL receptor-deficient mice

**DOI:** 10.1186/s12967-016-0972-z

**Published:** 2016-07-19

**Authors:** Daniele M. Guizoni, Gabriel G. Dorighello, Helena C. F. Oliveira, Maria A. Delbin, Marta H. Krieger, Ana P. Davel

**Affiliations:** Department of Structural and Functional Biology, Institute of Biology, University of Campinas-UNICAMP, P.O. Box 6109, Campinas, São Paulo, Brazil

**Keywords:** Aerobic exercise training, LDL receptor-deficient mice, Familial hypercholesterolemia, Endothelial dysfunction, Nitric oxide synthase, Hydrogen peroxide, Superoxide dismutase

## Abstract

**Background:**

Endothelial dysfunction associated with hypercholesterolemia is an early event in atherosclerosis characterized by redox imbalance associated with high superoxide production and reduced nitric oxide (NO) and hydrogen peroxide (H_2_O_2_) production. Aerobic exercise training (AET) has been demonstrated to ameliorate atherosclerotic lesions and oxidative stress in advanced atherosclerosis. However, whether AET protects against the early mechanisms of endothelial dysfunction in familial hypercholesterolemia remains unclear. This study investigated the effects of AET on endothelial dysfunction and vascular redox status in the aortas of LDL receptor knockout mice (LDLr^−/−^), a genetic model of familial hypercholesterolemia.

**Methods:**

Twelve-week-old C57BL/6J (WT) and LDLr^−/−^ mice were divided into sedentary and exercised (AET on a treadmill 1 h/5 × per week) groups for 4 weeks. Changes in lipid profiles, endothelial function, and aortic NO, H_2_O_2_ and superoxide production were examined.

**Results:**

Total cholesterol and triglycerides were increased in sedentary and exercised LDLr^−/−^ mice. Endothelium-dependent relaxation induced by acetylcholine was impaired in aortas of sedentary LDLr^−/−^ mice but not in the exercised group. Inhibition of NO synthase (NOS) activity or H_2_O_2_ decomposition by catalase abolished the differences in the acetylcholine response between the animals. No changes were noted in the relaxation response induced by NO donor sodium nitroprusside or H_2_O_2_. Neuronal NOS expression and endothelial NOS phosphorylation (Ser1177), as well as NO and H_2_O_2_ production, were reduced in aortas of sedentary LDLr^−/−^ mice and restored by AET. Incubation with apocynin increased acetylcholine-induced relaxation in sedentary, but not exercised LDLr^−/−^ mice, suggesting a minor participation of NADPH oxidase in the endothelium-dependent relaxation after AET. Consistent with these findings, Nox2 expression and superoxide production were reduced in the aortas of exercised compared to sedentary LDLr^−/−^ mice. Furthermore, the aortas of sedentary LDLr^−/−^ mice showed reduced expression of superoxide dismutase (SOD) isoforms and minor participation of Cu/Zn-dependent SODs in acetylcholine-induced, endothelium-dependent relaxation, abnormalities that were partially attenuated in exercised LDLr^−/−^ mice.

**Conclusion:**

The data gathered by this study suggest AET as a potential non-pharmacological therapy in the prevention of very early endothelial dysfunction and redox imbalance in familial hypercholesterolemia via increases in NO bioavailability and H_2_O_2_ production.

## Background

Familial hypercholesterolemia (FH) is a genetic disorder characterized by high concentrations of low-density lipoprotein (LDL), resulting in premature development of atherosclerosis and its vascular complications [1]. Endothelial dysfunction has been described as an important early marker of atherosclerosis preceding atheroma formation and is characterized by impaired vasodilatation [2]. Therefore, non-pharmacological strategies preventing the onset of endothelial dysfunction may protect against the acceleration of atherosclerosis in FH.

LDL receptor knockout (LDLr^−/−^) mice are a genetic model of hypercholesterolemia that mimics human FH [3]. It is accepted that LDLr^−/−^ mice fed a chow diet develop small lesions in the aortic root and impaired endothelium-dependent relaxation in the thoracic aorta [4, 5], despite lacking atherosclerotic plaques throughout the descending aorta [6–8]. In this model of genetic hypercholesterolemia, endothelial dysfunction is associated with impaired hydrogen peroxide (H_2_O_2_) production [4]. Similar to the effects of endothelial NOS (eNOS)-derived nitric oxide (NO), H_2_O_2_ production by neuronal nitric oxide synthase (nNOS) contributes to endothelium-dependent relaxation in the mouse aorta [9]. Furthermore, increased NADPH oxidase Nox2-derived superoxide anion production, together with a reduction in NO bioavailability, is often found to contribute to the development of atherosclerosis in humans and experimental models [10–12]. Thus, reactive oxygen species (ROS) participate in the pathogenesis of atherosclerotic disease via multiple pathways.

It is well established that aerobic exercise training (AET) promotes vascular beneficial effects, mitigating the risk of cardiovascular disease [2]. AET has been demonstrated to improve oxidative stress and endothelium-dependent relaxation, delaying the progression of vascular injury in atherosclerosis-prone model apolipoprotein E knockout mice (ApoE^−/−^) fed a high-fat and cholesterol diet [11, 13]. Atherogenic diets are often associated with metabolic disorders, including obesity and type 2 diabetes. Previous studies have demonstrated that treadmill AET improves endothelial dependent-relaxation [14], oxidative stress [15], NO release and nNOS expression [16] in obese non-atherosclerosis-prone rats. However, the potential beneficial effects of AET in the early stages of atherosclerosis, independent of atherogenic diet-induced confounding factors, are unclear. Recently, Langbein et al. [5] demonstrated that voluntary physical activity was not able to improve endothelial dysfunction in aortas from LDLr^−/−^ mice fed a standard or high-fat diet. However, it is still unknown whether or not an AET program can modulate endothelial function and vascular redox status in aortas from LDLr^−/−^ mice.

Therefore, in the present study, we investigated the effect of treadmill AET on impaired endothelium-induced relaxation in LDLr^−/−^ mice, focusing on the effects of vascular production of the superoxide anion, NO, and H_2_O_2_.

## Methods

### Animals

All experimental protocols were approved (protocol number: 3420-1) by the Ethics Committee on Animal Use of the University of Campinas (CEUA-UNICAMP, Campinas-SP, Brazil) and conformed to the ethical principles for animal experimentation adopted by the Brazilian Society of Laboratory Animal Science (SBCAL/COBEA). LDL receptor knockout (LDLr^−/−^) mice and wild-type (WT) mice (C57BL/6J) were purchased from the Jackson Laboratory; the strains are now maintained by breeding in the Multidisciplinary Center for Biological Research (CEMIB) of UNICAMP with genotypic control. The mice were housed at a constant room temperature (22–24 °C) and light cycle (12:12 h light:dark). All mice were fed a chow diet. Food and water were provided ad libitum to all animals.

WT and LDLr^−/−^ mice (12-week-old) were divided into four experimental groups: sedentary WT (WT S, n = 20) and exercised WT (WT Ex, n = 20) and sedentary LDLr^−/−^ (LDLr^−/−^ S, n = 20) and exercised LDLr^−/−^ (LDLr^−/−^ Ex, n = 20). The sample size was defined based on the variability of the different analyzes using the GraphPad Statmate version 2.0.

### Aerobic exercise protocol

Animals were trained on a treadmill with individual lanes designed for small animals, without electrical stimulation (Gesan, Sao Paulo-SP, Brazil). One week before starting the AET program, the mice were subjected to a treadmill adaptation protocol in an attempt to minimize potential stress; this consisted of daily runs beginning at 10 m/min for 10 min on the first day and was progressively increased to 10 m/min for 30 min. Only the animals that adapted to the treadmill were used in the present study. After 4 days of adaptation, the intensity of the exercise training was determined according to an acute incremental exercise test on the treadmill [17], where the intensity of exercise was increased by 3.3 m/min (5–38.3 m/min) every 3 min at a 0 % gradient until the point of exhaustion (defined as an inability to maintain running speed). The maximal speed was used to calculate the percentage corresponding to moderate intensity (60–70 % of maximal speed). At the beginning of the training program, the duration and speed started at 10 m/min for 30 min and were progressively increased to 60 min at a speed of 15.0–16.6 m/min, 5 days/week for 4 weeks, at a 0 % gradient. All animals were trained between 7:00 and 10:00 a.m.

To evaluate the effectiveness of the training program, sedentary and exercised mice were subjected to an acute incremental exercise test on the treadmill during the last week of the study. This test provided the total distance, total time, and maximal speed run for each animal.

### Serum biochemical analysis

At the end of the training protocol, 48 h after the last exercise session and after 12 h of fasting, blood samples were collected from the tail vein and blood glucose was measured using a hand-held glucometer (Accu-Chek Advantage, Roche Diagnostics, Sao Paulo, Brazil). Immediately thereafter, the mice were anesthetized (5 g/kg urethane, i.p.), and blood samples were obtained by cardiac puncture and centrifuged (8000*g*, for 15 min); serum supernatants were subsequently collected for biochemical analysis. Total cholesterol (CHOL) and triglyceride (TG) levels were measured using standard commercial kits (Chod-Pap, Roche Diagnostic GmbH, Mannheim, Germany); the absorbance for CHOL and TG was measured at 492 nm.

### Vascular reactivity study

Thoracic aortas were cut into segments (2 mm in length), free of adipose and connective tissue, and then mounted in an isolated tissue chamber containing Krebs-Henseleit solution (in mM: 118 NaCl, 4.7 KCl, 25 NaHCO_3_, 2.5 CaCl_2_-2H_2_O, 1.2 KH_2_PO_4_, 1.2 MgSO_4_-7H_2_O, 11 glucose, and 0.01 EDTA) and gassed with 95 % O_2_ and 5 % CO_2_. The rings were maintained at a resting tension of 0.5 g at 37 °C, pH = 7.4, as previously described [18]. Isometric tension was recorded using an isometric force transducer (MLT0420, ADInstruments, Sydney, Australia) connected to an acquisition PowerLab 8/30 system for tension recordings (LabChart 7, ADInstruments). After a 60 min equilibration period, the aortic rings were exposed to 125 mM KCl to determine the maximal tension. After washout, relaxation concentration–response curves to acetylcholine (ACh, 1 nM–30 µM, Sigma-Aldrich, Saint Louis, MO, USA), NO-donor sodium nitroprusside (10 pM–3 µM, Sigma-Aldrich, Saint Louis, MO, USA), or exogenous H_2_O_2_ (1 µM–3 mM, Sigma-Aldrich, Saint Louis, MO, USA) were generated for the aortic rings contracted with U46619 (Enzo Life Sciences, Farmingdale, NY, USA) until 50–70 % of maximum contraction with 125 mM KCl was reached. Some aortic rings were incubated for 30 min with the nonselective NOS inhibitor N-nitro-l-arginine methyl ester (L-NAME, 100 µM, Sigma-Aldrich, Saint Louis, MO, USA), the NADPH oxidase inhibitor apocynin (30 µM, Sigma-Aldrich, Saint Louis, MO, USA), the SOD inhibitor diethyldithiocarbamate (DETCA, 1 mM, Sigma-Aldrich, Saint Louis, MO, USA), the nNOS inhibitor 7-nitroindazole (7-NI, 100 µM, Sigma-Aldrich, Saint Louis, MO, USA), or catalase (800 U/ml, Sigma-Aldrich, Saint Louis, MO, USA) before the relaxation response curves to ACh were generated.

### Western blotting analyses

Protein extracts were obtained from isolated thoracic aortas homogenized in RIPA lysis buffer (Merck Millipore, Billerica, MA, USA) containing phenylmethylsulfonyl fluoride (1 mM PMSF), Na_3_VO_4_ (1 mM) and a protease inhibitor cocktail (2 μL/mL PIC). To investigate the eNOS dimer:monomer ratio, aortas were lysed in buffer (50 mmol/L Tris–HCl pH = 8.0; 0.2 % Nonidet P-40; 180 mmol/L NaCl; 0.5 mmol/L EDTA; 25 mmol/L phenylmethylsulphonyl fluoride; 0.1 mmol/L dithiothreitol; and protease inhibitors). Protein samples (40 µg) were electrophoretically separated by SDS-PAGE (4–15 % Mini-Protean TGX, BioRad, CA, USA) at room temperature. To analyze eNOS dimerization, non-boiled samples (50 µg) were resolved by 6 % SDS-PAGE at 4 °C. Next, the proteins were transferred onto polyvinylidene fluoride (PVDF) membranes (GE Healthcare, Little Chalfont, Buckinghamshire, UK) overnight at 4 °C using a Mini Trans-Blot Cell System (Bio-Rad, Hercules, CA, USA), as previously described [18]. The membranes were subsequently blocked for 90 min at room temperature with 5 % albumin in Tris-buffer (10 mM Tris, 100 mM NaCl and 0.1 % Tween 20) and then incubated overnight at 4 °C with the following primary antibodies: anti-eNOS (1:1000; BD Transduction, Franklin Lakes, NJ, USA), anti-p-eNOS Ser1177 (1:500; Cell Signaling), anti-nNOS (1:1000; Santa Cruz Biotechnology, CA, USA), anti-CuZn-SOD (1:1000, Sigma-Aldrich, Saint Louis, MO, USA), anti-Mn-SOD (1:1000, Enzo Life Sciences, Farmingdale, NY, USA), anti-EC-SOD (1:500, Enzo Life Sciences, Farmingdale, NY, USA), anti-Nox2 (1:1000 Millipore, Billerica, MA, USA) and anti-catalase (1:1000, Sigma-Aldrich, Saint Louis, MO, USA).

Membranes containing non-boiled samples were incubated with anti-eNOS (1:750, BD Transduction, Franklin Lakes, NJ, USA). α-actin (primary antibody 1:5000, Abcam, Cambridge, UK) was used to normalize the expression of the evaluated proteins in each sample. After washing with Tris-buffer, the membranes were incubated for 90 min with a peroxidase-conjugated IgG antibody, according to each primary antibody used. Protein expression was detected using the Pierce ECL (Electrochemiluminescence) kit. Western blotting substrate (Thermo Scientific, Rockford, IL, USA) was used for chemiluminescent detection using Image Quant LAS 4000 (GE Healthcare, NY, USA) hardware and software, according to the manufacturer’s instructions. The intensity of the blots was quantified using ImageJ 1.46p software (National Institutes of Health, Bethesda, MD, USA). Next, eNOS dimerization of the dimer (260 kDa) to monomer (130 kDa) ratio was calculated.

### NO production

NO production was evaluated using the NO-sensitive fluorescent dye 4,5-diaminofluorescein diacetate (DAF-2A), as previously described [19]. Thoracic aortic rings (approximately 3 mm in length) were embedded in a cryoprotectant freezing medium (Tissue-Tek^®^ O.C.T compound 4583, Torrance, CA, USA) to obtain transverse sections (10 μm) using a cryostat. The slices were equilibrated for 10 min in a phosphate buffer (PB 0.1 mM, pH = 7.4) containing CaCl_2_ (0.45 mM) at 37 °C. Next, DAF-2A (8 μM) was topically applied to the slices, and they were maintained for 30 min in a light-protected humidified chamber. Digital images were obtained using a microscope (Eclipse Ti-S, Nikon, Tokyo, Japan) equipped for epifluorescence with a standard fluorescein filter with a 20 × objective. The images were analyzed using ImageJ 1.46p software by measuring the mean optical density of the fluorescence.

### H_2_O_2_ production

Extracellular H_2_O_2_ was detected using Amplex Red [20]. To achieve this, four aortic rings (1.5 mm) were placed in a well of a 96-well dark plate and incubated with Amplex Red (10 μM) and horseradish peroxidase (0.2 U/mL) for 60 min at 37 °C in Krebs–Ringer’s phosphate glucose buffer (in mM: 145 NaCl, 5.7 sodium phosphate, 4.86 KCl, 0.54 CaCl_2_, 1.22 MgSO_4_, and 5.5 glucose) protected from light. Fluorescence was detected at 590 nm using an excitation of 530 nm every 5 min for 60 min. H_2_O_2_ was expressed as fluorescence per minute. In some experiments, catalase (200 U/mL) was added to the tissue samples.

### Superoxide anion detection

Hydroethidine was used as an oxidative fluorescent dye [18]. Transverse aortic sections (10 µm) obtained in a cryostat were incubated at 37 °C for 10 min with phosphate buffer. Hydroethidine (5 µM) was topically applied to each tissue section, and the slides were incubated in a light-protected, humidified chamber at 37 °C for 30 min. Some aortic slices were incubated with phosphate buffer containing the membrane-permeable superoxide dismutase (SOD) mimetic Mn(III) tetrakis (1-methyl-4-pyridyl) porphyrin pentachloride (MnTMPyP, 25 µM, Calbiochem, San Diego, CA, USA), apocynin (30 µM, 30 min), or vehicle (deionized water; time controls). Images were obtained using an optical microscope (Eclipse Ti-S, Nikon, Tokyo Japan) equipped with a rhodamine filter and camera (DS-U3, Nikon, Tokyo, Japan), using a 40× objective. The images were analyzed using ImageJ 1.46p software by measuring the mean optical density of the fluorescence.

### Statistical analysis

The results are expressed as the mean ± SEM. Data were analyzed using two-way ANOVA followed by Bonferroni post-test using GraphPad Prism 5.0 software. Values of p < 0.05 were considered statistically significant.

## Results

### Physical performance, body weight and serum biochemical parameters in sedentary and trained mice

All groups presented similar responses to the acute incremental exercise test before the start of AET (Table 1). Exercised mice exhibited increased total time (min, approximately 50 %), total distance (m, approximately 100 %) and maximal speed (m/min, approximately 35 %) values in the final incremental exercise test compared with the respective sedentary groups (Table 1). These results confirmed the effectiveness of the AET protocol in improving physical performance in both the WT Ex and the LDLr^−/−^ Ex groups. No differences were observed between the WT and LDLr^−/−^ groups after training (Table 1).

**Table 1:** Incremental exercise test performed before (initial) and after (final) 4-week sedentary (S) or exercise-trained (Ex) wild-type (WT) and LDL receptor knockout mice (LDLr^−/−^)

	Initial				Final			
	WT S	WT Ex	LDLr^−/−^ S	LDLr^−/−^ Ex	WT S	WT Ex	LDLr^−/−^ S	LDLr^−/−^ Ex
Time (min)	18 ± 0.5	18 ± 0.9	16 ± 0.6	17 ± 0.7	17 ± 0.6	26 ± 1.2*	17 ± 0.7	26 ± 1.3^#^
Distance (m)	243 ± 10	250 ± 22	208 ± 12	225 ± 15	223 ± 14	459 ± 38*	220 ± 16	486 ± 42^#^
Max speed (m/min)	24 ± 0.5	25 ± 0.8	22 ± 0.6	23 ± 0.8	23 ± 0.7	31 ± 1.5*	22 ± 0.8	32 ± 1.2^#^

Data are mean ± SEM. The number of animals per group was: WT S (n = 20); WT Ex (n = 20); LDLr^−/−^ S (n = 20); LDLr^−/−^ Ex (n = 20)

Two-way ANOVA: * p < 0.05 vs. WT S; ^#^ p < 0.05 vs. LDLr^−/−^ S

Body weights were similar in all groups at the initial time of the study. After the protocol, AET decreased body weight in both the WT Ex (8 %) and the LDLr^−/−^ Ex (12 %) groups compared with the WT S and the LDLr^−/−^ S groups (Table 2). The LDLr^−/−^ S mice exhibited approximately threefold higher CHOL and TG levels than the WT S mice. AET did not modify CHOL or TG levels in either the WT Ex or the LDLr^−/−^ Ex groups (Table 2). Fasting glucose levels were not different among the groups (Table 2).

**Table 2:** Body weight and plasma biochemical parameters in sedentary (S) and exercise-trained (Ex) wild-type (WT) and LDLr knockout mice (LDLr^−/−^)

	WT S	WT Ex	LDLr^−/−^ S	LDLr^−/−^ Ex
Initial body weight (g)	25 ± 0.2 (20)	25 ± 0.5 (20)	24 ± 0.6 (20)	24 ± 0.2 (20)
Final body weight (g)	26 ± 0.3 (20)	24 ± 0.4* (20)	26 ± 0.8 (20)	23 ± 0.5^#^ (20)
CHOL (mg/dL)	82 ± 4 (13)	92 ± 6 (9)	262 ± 14* (16)	279 ± 15^$^ (14)
TG (mg/dL)	54 ± 4 (11)	49 ± 6 (7)	151 ± 11* (11)	147 ± 9^$^ (10)
Glucose (mg/dL)	110 ± 6 (7)	90 ± 5 (7)	114 ± 11 (7)	107 ± 6 (7)

Data are mean ± SEM. The number of animals is in parentheses

*CHOL* total cholesterol; *TG* triacylglycerol

Two-way ANOVA: * p < 0.05 vs. WT S; ^#^ p < 0.05 vs. LDLr^−/−^ S; ^$^ p < 0.05 vs. WT Ex

### Aerobic exercise training prevented endothelial dysfunction in the thoracic aorta of LDLr^−/−^ mice

**Fig. 1:**
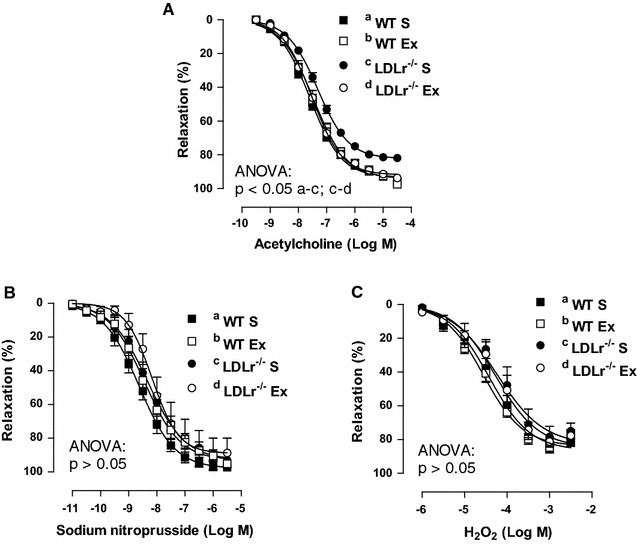
Concentration-response curves to acetylcholine (**A**), to sodium nitroprusside (**B**) and to hydrogen peroxide (H_2_O_2_, **C**) in thoracic aortic rings from sedentary (S) and exercise-trained (Ex) wild-type (WT) and LDLr knockout mice (LDLr^−/−^). Data are mean ± SEM (n = 5–14 per group). 2-way ANOVA

The aorta of LDLr^−/−^ S mice showed reduced endothelium-dependent relaxation in response to ACh compared with that of WT S mice, which was fully prevented by AET (Fig. 1A). No significant differences in sodium nitroprusside-induced relaxation or H_2_O_2_-induced relaxation were observed between the experimental groups, suggesting that neither LDLr deficiency or 4 weeks of AET interferes with aortic smooth muscle sensitivity to NO and H_2_O_2_ (Fig. 1B, C). Endothelial dysfunction was observed in LDLr^−/−^ S mice despite the lack of evidence of fat deposition in the descending thoracic aorta (data not shown).

### Aerobic exercise training improves affected NO and H_2_O_2_ production in LDLr^−/−^ mice

**Fig. 2:**
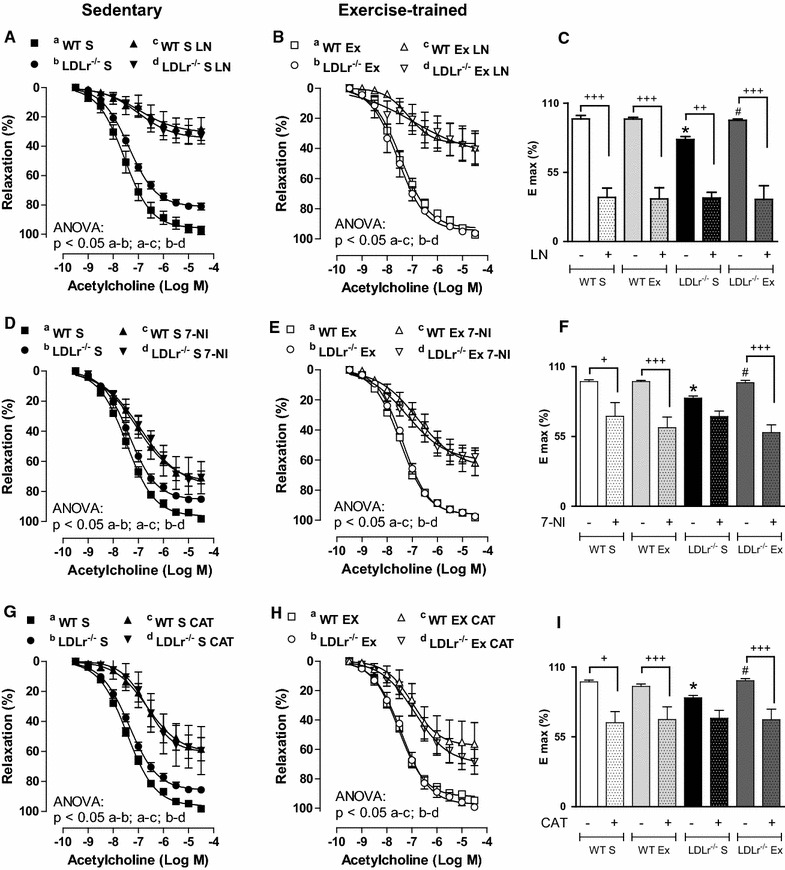
Concentration-response curves to acetylcholine before and after incubation with L-NAME (LN, **A** and **B**), 7-Nitroindazole (7-NI, **D** and **E**) or catalase (CAT, **G** and **H**) in thoracic aortic rings from sedentary (S) and exercise-trained (Ex) wild-type (WT) and LDLr knockout mice (LDLr^−/−^). *Bar graphs* show the acetylcholine maximal vasodilator effect (E_max_) in the presence or absence of L-NAME (**C**), 7-NI (**F**) and CAT (**I**). ANOVA: * p < 0.05 vs. WT S;^#^ p < 0.05 vs. LDLr^−/−^ S; + indicates p < 0.05, ++ indicates p < 0.001, and +++ indicates p < 0.0001 vs. without incubation ring. Data are mean ± SEM (n = 5–8 per group)

As ACh stimulates endothelial eNOS-derived NO and nNOS-derived H_2_O_2_ production in the mouse aorta [9], we incubated aortic rings with the non-selective NOS inhibitor L-NAME or with the nNOS inhibitor 7-NI. L-NAME reduced ACh-induced relaxation in all groups (Fig. 2A, B), abolishing the differences between WT S and LDLr^−/−^ S mice (Fig. 2A). The effect of L-NAME in reducing the maximum vasodilator effect (E_max_) of ACh was less significant in LDLr^−/−^ S than in WT S mice; AET reversed this effect (Fig. 2A–C). 7-NI significantly reduced ACh-induced relaxation, but to a lesser extent than L-NAME, demonstrating the specific role of nNOS in this process (Fig. 2D, E). In contrast to the other groups, the E_max_ of ACh in the aortas of LDLr^−/−^S mice was not modified by the nNOS inhibitor (Fig. 2F). Together, these data suggest impairment of the NOS component of ACh-induced relaxation in the aortas from LDLr^−/−^ S, which is prevented by AET. No differences were observed regarding the effects of L-NAME and 7-NI between the WT Ex and LDLr^−/−^ Ex mice (Fig. 2B, E).

To evaluate the participation of H_2_O_2_ in endothelium-dependent relaxation, some aortic rings were incubated with catalase, which decomposes H_2_O_2_. Catalase reduced the vasodilatory effect of ACh in all groups, abolishing the differences among them (Fig. 2G, H). The E_max_ graph in Fig. 2I shows that the inhibitory effect of catalase was less prominent in LDLr^−/−^ S than in WT S mice, while no difference was found for LDLr^−/−^ Ex compared with WT Ex mice (Fig. 2I). These data suggest that AET attenuates the effect of H_2_O_2_ on vasodilation in LDLr-deficient mice.

**Fig. 3:**
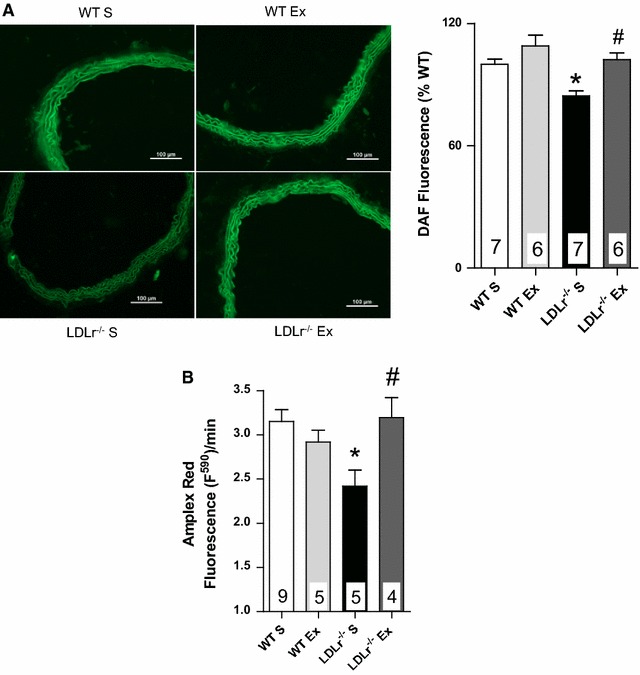
Representative fluorographs (**A**, *left panel*) and quantitative analysis (**A**, *bar graphic right*) of NO production evaluated by DAF-2A fluorescence in transverse sections of thoracic aorta. DAF-2A fluorescence is expressed as the percentage of intensity per vessel area obtained in WT S group (bar scale = 100 μm). The *bar graph* (**B**) represents the fluorescence curve slope to Amplex Red estimating the production of H_2_O_2_ per minute in aortic rings from sedentary (S) and exercise-trained (Ex) wild-type (WT) and LDLr knockout mice (LDLr^−/−^). Data are mean ± SEM. Two-way ANOVA: * p < 0.05 vs. WT S; ^$^ p < 0.05 vs. WT Ex; ^#^ p < 0.05 vs. LDLr^−/−^ S. Numbers into the *bars* represent N of animals used in each group

Regarding the bioavailability of NO, DAF-2A fluorescence analysis revealed a significant reduction in NO levels in the aortas of LDLr^−/−^ S mice compared with the aortas of WT S mice (Fig. 3A). In addition, H_2_O_2_ production evaluated by Amplex Red fluorescence was also significantly reduced in the aortas of LDLr^−/−^ S mice (Fig. 3B). AET prevented the reductions in both NO and H_2_O_2_ in LDLr^−/−^ Ex mice but exerted no significant effects in WT Ex mice (Fig. 3A, B). The presence of catalase significantly reduced Amplex Red fluorescence, confirming H_2_O_2_ as the main factor evaluated using this technique (data not shown).

**Fig. 4:**
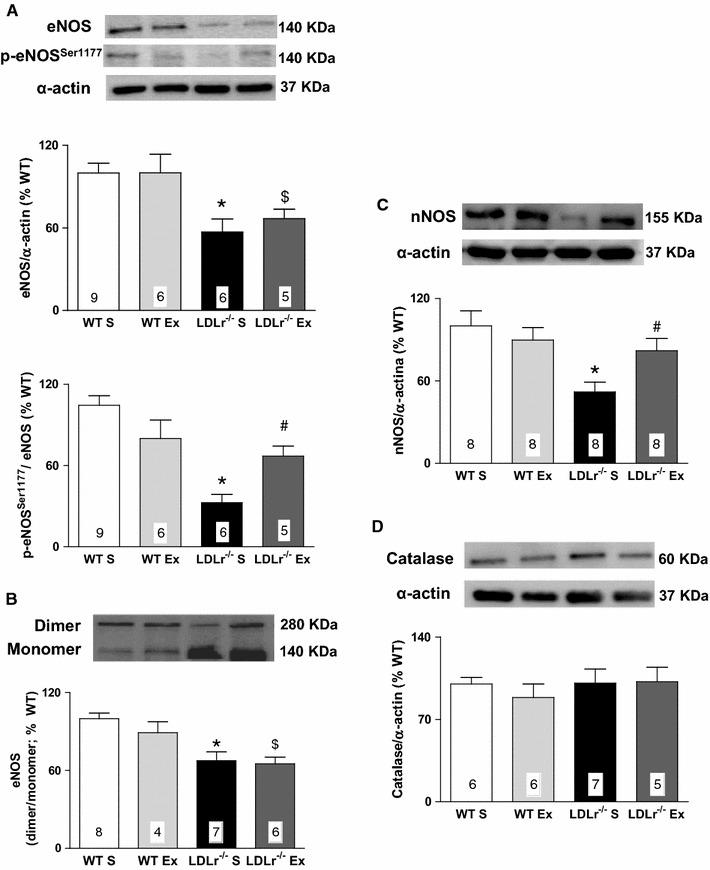
Representative blots (*top*) and quantitative protein expression (*bottom*) of total and phosphorylated eNOS (p-eNOS) at Ser1177 (**A**); dimerized eNOS (**B**); total nNOS (**C**) and catalase (**D**) in aorta from sedentary (S) and exercise-trained (Ex) wild-type (WT) and LDLr knockout mice (LDLr^−/−^). Proteins expression were normalized to α-actin content in each sample, and protein expression of p-eNOS were normalized to total eNOS expression. eNOS dimerization was expressed as a ratio of dimer:monomer band intensity. The results were expressed as the percentage of the protein expression values obtained in WT S group. Data are mean ± SEM. Two-way ANOVA: * p < 0.05 vs. WT S; ^$^ p < 0.05 vs. WT Ex; ^#^ p < 0.05 vs. LDLr^−/−^ S. Numbers into the *bars* represent N of animals used in each group

The protein expression of total eNOS, Ser1177-phosphorylated eNOS, eNOS dimer, and total nNOS was significantly reduced in the aortas of the LDLr^−/−^ S group, compared with those of WT S group (Fig. 4A–C). AET restored the expression of Ser1177-phosphorylated eNOS and total nNOS in LDLr^−/−^ Ex mice (Fig. 4A, C). The decreased eNOS dimerization in the aortas of LDLr^−/−^ mice was not modified by exercise (Fig. 4B). In addition, the protein expression of catalase did not differ among the groups (Fig. 4D).

### Aerobic exercise training reduces the synthesis and increases the degradation of the superoxide anion

**Fig. 5:**
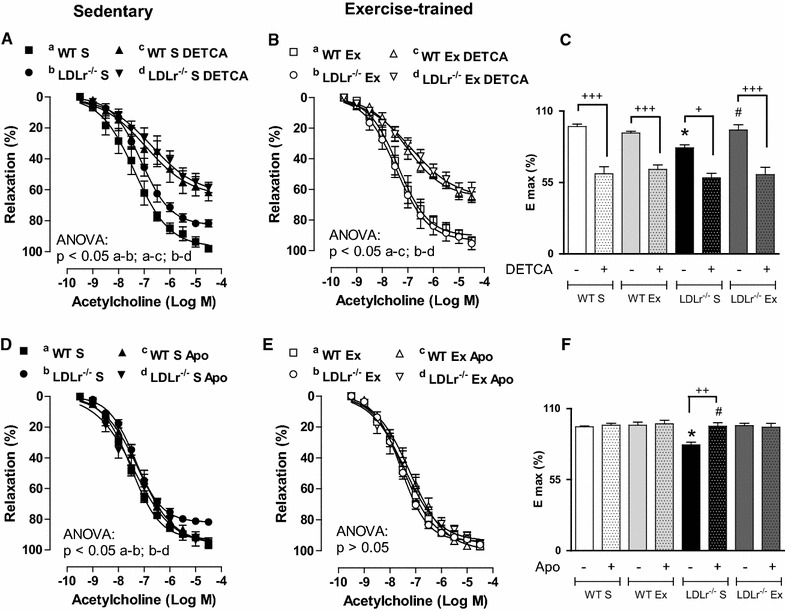
Concentration-response* curves* to acetylcholine before and after incubation with diethyldithiocarbamate (DETCA, **A** and **B**), or apocynin (Apo, **D** and **E**) in thoracic aortic rings from sedentary (S) and exercise-trained (Ex) wild-type (WT) and LDLr knockout mice (LDLr^−/−^). *Bar graphs* show the acetylcholine maximal vasodilator effect (E_max_) in the presence or absence of DETCA (**C**) and Apo (**F**). ANOVA: * p < 0.05 vs. WT S;^#^ p < 0.05 vs. LDLr^−/−^ S; + indicates p < 0.05, ++ indicates p < 0.001, and +++ indicates p < 0.0001 vs. without incubation rings. Data are mean ± SEM (n = 5–8 per group)

The participation of superoxide in the endothelial dysfunction of LDLr^−/−^ mice was evaluated in rings incubated with the SOD inhibitor DETCA or the NADPH oxidase inhibitor apocynin. DETCA reduced the ACh-induced relaxation in all groups, indicating the importance of endogenous CuZn-SODs activity to endothelium-dependent vasorelaxation in the mouse aorta (Fig. 5A, B). After SOD inhibition, no differences were found among the groups (Fig. 5A, B). Furthermore, the reduction in E_max_ to ACh induced by DETCA was less significant in LDLr^−/−^ S than in WT S mice; AET reversed this effect (Fig. 5C). These data revealed that the impaired SOD effect in the endothelium-dependent relaxation of LDLr-deficient mice is prevented by AET (Fig. 5A–C). Incubation with apocynin increased ACh-induced relaxation in LDLr^−/−^ S mice, normalizing this response (Fig. 5D). No significant effect for apocynin was observed in the aortic rings of the WT S, WT Ex, and LDLr^−/−^ Ex groups (Fig. 5D–F), indicating that AET prevents against NADPH oxidase-induced endothelial dysfunction in the aorta of LDL-deficient mice.

**Fig. 6:**
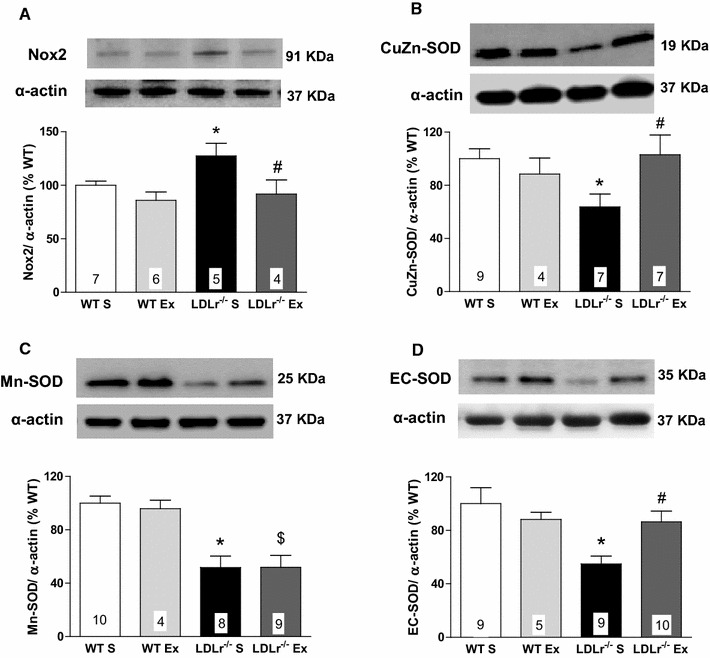
Representative blots (*top*) and quantitative protein expression (*bottom*) of Nox2 subunit of NADPH oxidase (**A**); CuZn-SOD (**B**); Mn-SOD (**C**) and EC-SOD (**D**) in aorta from sedentary (S) and exercise-trained (Ex) wild-type (WT) and LDLr knockout mice (LDLr^−/−^). Proteins expression were normalized to α-actin content in each sample. The results were expressed as the percentage of the protein expression values obtained in WT S group. Data are mean ± SEM. Two-way ANOVA: * p < 0.05 vs. WT S; ^$^ p < 0.05 vs. WT Ex; ^#^ p < 0.05 vs. LDLr^−/−^ S. Numbers into the *bars* represent N of animals used in each group

Next, we evaluated the expression of proteins related to the synthesis (NADPH oxidase Nox2) and degradation (SOD isoforms) of the superoxide anion. The NADPH oxidase Nox2 was up-regulated, while the protein expression of Cu/Zn-, Mn-, and EC-SOD were reduced in the aorta of the LDLr^−/−^ S group (Fig. 6A–D). AET normalized the alterations of Nox2 (Fig. 6A), Cu/Zn- (Fig. 6B), and EC-SOD (Fig. 6D) with respect to protein expression in LDLr^−/−^ Ex mice.

**Fig. 7:**
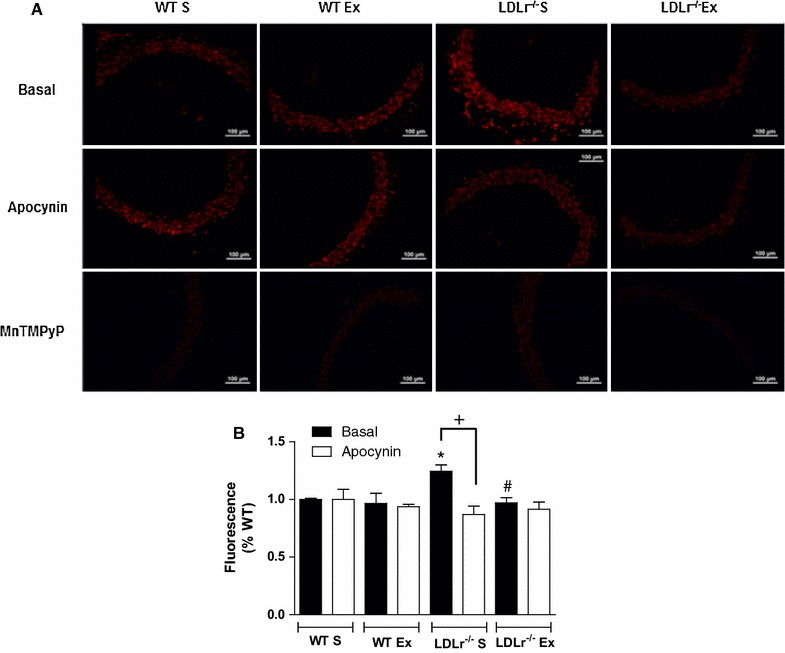
Representative fluorographs (**A**) and quantitative analysis of reactive oxygen species production in transverse sections of aorta (**B**) evaluated by the ethidium-bromide-positive nuclei under basal conditions and after incubation with apocynin (30 mM), or with Mn(III) tetrakis1-methyl-4-pyridyl porphyrin pentachloride (MnTMPyP, 25 µM).* Bar scale* 100 μm. Sedentary and exercised (Ex) wild-type (WT) and LDLr knockout mice (LDLr^−/−^). Data are mean ± SEM (n = 4–15 per group). 2-way ANOVA: * p < 0.05 vs. WT S; ^#^ p < 0.05 vs. LDLr^−/−^ S; + p < 0.05 apocynin vs. basal

According to the above results, we evaluated vascular superoxide anion production. Increased fluorescence emitted by hydroethidine-derived oxidation products revealed increased ROS formation in aortic slices from LDLr^−/−^ S compared with those from WT S mice, which was reversed by apocynin or AET (Fig. 7A, B). Incubation with the SOD mimetic MnTMPyP dramatically reduced the fluorescence of hydroethidine in all groups (Fig. 7A), suggesting the superoxide anion as the major vascular ROS up-regulated by LDLr-deficiency and normalized by AET.

## Discussion

This study investigated the protective mechanisms of AET in preventing endothelial dysfunction during the early phase of atherosclerotic disease in LDLr-deficient mice. We found that 4 weeks of AET fully reversed the endothelial vasodilatory dysfunction observed in the aortas of LDLr^−/−^ mice by improving NO bioavailability and H_2_O_2_ production. AET increased the phosphorylation of eNOS and aortic expression of nNOS in LDLr^−/−^ mice, suggesting that an up-regulation of the signaling pathways of constitutive isoforms of NOS should be involved in the vascular effects of AET. Furthermore, the improvement of the vascular redox state in exercised-LDLr^−/−^ mice was associated with the up-regulation of the antioxidant defense exerted by SOD and the down-regulation of NADPH oxidase-derived superoxide production. The vascular benefits conferred by AET occurred despite persistent hypercholesterolemia. Therefore, our data revealed that moderate AET may be an effective non-pharmacological therapy for the early prevention of vascular complications of artery disease in FH.

Endothelial dysfunction has been described in the aorta of LDLr-deficient mice fed a standard diet at 12–24 weeks of age [4, 5]. Consistent with the findings of these previous studies, we observed impaired ACh-induced relaxation in the aortas of 16-week-old LDLr^−/−^ mice. Previous studies have demonstrated that long periods of training (12 weeks) reduced preexisting atherosclerotic lesion area in arteries from LDLr^−/−^ mice [21] and ApoE^−/−^ mice [22] fed atherogenic diets. Because endothelial dysfunction is an important predictor of atherosclerosis development and future cardiovascular events, we evaluated the effect of AET in controlling this risk factor in a genetic background of hypercholesterolemia.

Here, we report for the first time that 4 weeks of a treadmill-based AET program fully prevented the early endothelial dysfunction found in the thoracic aortas of LDLr^−/−^ mice without atherosclerotic plaques. In contrast, Langbein et al. [5] found that 20 weeks of voluntary running did not ameliorate endothelial dysfunction in LDLr^−/−^ mice fed standard or high-fat diets. Importantly, these authors observed a decrease in mice spontaneous activity over time, which may cause a lack-of-exercise effect on endothelial function. Here, the effectiveness of the training program was confirmed by the significant increases in total time, total distance, and maximal speed reached during the incremental test performed after the 4-week training protocol in WT Ex mice and LDLr^−/−^ Ex mice.

It has been demonstrated that acute aerobic exercise is sufficient to activate eNOS in healthy endothelial cells of the mouse aorta; this effect occurs via a signaling pathway dependent on AMP-activated protein kinase activation (AMPK), resulting in Ser1177 eNOS phosphorylation, without changes in total eNOS expression or in Ser635 and Thr495 eNOS phosphorylation [23]. In the present study, we demonstrated that 4 weeks of AET reversed the reduction in Ser1177 eNOS phosphorylation, normalizing the levels of NO in the aortas of LDLr^−/−^ mice to levels similar to those found in WT animals, without increasing the dimerization of eNOS. A similar mechanism was found in the left internal mammary arteries of 4-week exercise-trained patients with stable coronary artery disease [24]. Reduced eNOS dimerization may not necessarily be associated with eNOS uncoupling as other mechanisms are involved in electron transfer in eNOS domains [25]. Because endothelium-derived NO plays a protective role in the vasculature by inhibiting vascular smooth muscle cell proliferation and by suppressing the adhesion of inflammatory cells and platelets [26], exercise activates an important protective mechanism against the progression of atherosclerosis in genetic hypercholesterolemia.

eNOS-derived NO and nNOS-derived H_2_O_2_ equally contribute to endothelium-dependent vasodilation of the mouse aorta [9]. In young LDLr^−/−^ mice (10 weeks old), increased H_2_O_2_ release may compensate for reduced eNOS expression in the aorta [27]. However, nNOS mRNA expression and H_2_O_2_ levels decreased with age [27] or in accelerated atherosclerosis [28]. H_2_O_2_ and nNOS are considered antiatherogenic factors [29, 30]. Gene deletion of nNOS in ApoE^−/−^ mice accelerates atherosclerotic plaque formation [29], while overexpression of Nox4 forming H_2_O_2_ reduces the expression of profibrotic and pro inflammatory markers inhibiting vascular remodeling in mice prone to atherosclerosis [30]. We found that reductions in the nNOS and H_2_O_2_ components of ACh-induced relaxation of the aorta in 16-week-old LDLr^−/−^ mice were reversed by AET. No changes in relaxation with H_2_O_2_ or in the expression of vascular catalase were observed among the strains. Furthermore, AET prevented reductions in H_2_O_2_ production and nNOS expression in the aortas of LDLr^−/−^ mice. This finding demonstrates the beneficial effects of AET in the maintenance of endothelial function via the up-regulation of nNOS-derived H_2_O_2_ in the genetic background of hypercholesterolemia. However, a limitation of this study is that we cannot exclude that impaired endothelial Nox4 activity/expression leading to a reduction in H_2_O_2_ production is involved in the endothelial dysfunction in LDLr^−/−^ mice and modulated by AET.

Recent studies have indicated the beneficial effects of exercise in increasing nNOS signaling in the cardiovascular system. Four weeks of treadmill exercise increased the expression and activity of eNOS and nNOS in the aorta and left ventricle of chronic heart failure rats, respectively [31]. Furthermore, 8 weeks of AET resulted in nNOS overexpression in endothelium-denuded mesenteric arteries, enhancing the participation of nitrergic perivascular innervation in spontaneously hypertensive rats [32] and obese rats [16]. For the first time, we demonstrated that an AET program can up-regulate the antiatherogenic eNOS-NO and nNOS-H_2_O_2_ pathways, which is associated with amelioration of endothelial function in a genetic model of hypercholesterolemia. However, whether or not AET could induce similar vascular signaling pathway in the later stages of atherosclerotic disease is still an opened question.

Reduced formation of H_2_O_2_ was associated with increased Nox2 expression in atherosclerosis, but not with Nox1 expression [30]. Judkins et al. [12] first demonstrated a role for NADPH oxidase Nox2 in enhancing vascular ROS production, thereby reducing NO bioavailability and increasing lesion development in ApoE^−/−^ mice. In addition, we noted up-regulation of Nox2 in the aortas of LDLr^−/−^ S mice. Similarly, inhibition of NADPH oxidase or superoxide anion scavengers reversed endothelial dysfunction and the oxidative stress detected by hydroethidine product fluorescence in the aorta of LDLr^−/−^ S mice, implicating Nox2-derived superoxide in endothelial dysfunction in this hypercholesterolemic strain. Treadmill AET reversed Nox2 up-regulation, as well as enhanced vascular NADPH oxidase-derived superoxide production. Our findings are consistent with the beneficial effects of 10-week AET normalizing Nox2 protein expression in the aortas of type 2 diabetic mice [33].

SOD isoforms represent a major defense against the inactivation of NO and peroxynitrite formation [34]. However, findings related to the expression of SODs in vessels of experimental models of atherosclerosis are controversial. EC-SOD activity and protein expression were found to be increased [35] or decreased [36] in ApoE^−/−^ mice, whereas CuZn- and Mn-SOD were not modified [36]. Differences may be due to the type of Western diet and/or the stage of development of the atherosclerotic lesion. In the present study, we noted a reduction in the protein expression of the three isoforms of SOD in the early stages of atherosclerosis in LDLr^−/−^ mice fed a standard diet. Inhibition of Cu–Zn dependent SODs activity (cytoplasmatic CuZn SOD and EC-SOD) with DETCA, a powerful copper chelating agent, exerted a smaller effect on ACh-induced relaxation in LDLr^−/−^ S mice compared with WT S mice. This finding serves as functional proof that down-regulation of Cu-dependent SOD isoforms is a mechanism involved in endothelial dysfunction in genetic hypercholesterolemia.

We observed that 4 weeks of treadmill AET normalized both cytoplasmic and extracellular CuZn-SODs expression, concomitant with normalization of the effects of DETCA on ACh-induced relaxation in the aortas of LDLr-deficient mice. These results demonstrated the up-regulation of CuZn-SODs as a vascular protective mechanism in endothelial dysfunction in hypercholesterolemia. AET has been reported to increase vascular expression of CuZn–SOD in healthy [37] and obese animals [14, 33]. However, the mechanism by which exercise enhances SOD expression in cardiovascular diseases is not clear, but Fukai et al. [38] have suggested that increased eNOS-derived NO is a feed-forward mechanism that up-regulates EC-SOD expression in adjacent vascular cells, which in turn increases NO bioavailability.

## Conclusion

Taken together, our results suggest that AET improves endothelial dysfunction in the aortas of LDLr-deficient mice via the up-regulation of NO bioavailability and H_2_O_2_ production. These effects are associated with the up-regulation of p-eNOS, nNOS, and SOD isoforms and reduced Nox2-derived superoxide anion production. The vasculo-protective effects of 4 weeks of AET were observed in the early stages of atherosclerosis and were independent of changes in the dyslipidemic profile. These data highlight AET as a potential early non-pharmacological therapy for the prevention of endothelial dysfunction and changes in vascular redox status in patients with FH.

## Abbreviations

7-NI: 7-nitroindazole
ACh: acetylcholine
AET: aerobic exercise training
AMPK: 5′ adenosine monophosphate-activated protein kinase
ANOVA: analysis of variance
ApoE^−/−^: apolipoprotein E knockout mice
CAT: catalase
CEMIB: Multidisciplinary Center for Biological Research
CHOL: total cholesterol
CO_2_: carbon dioxide
Cu/Zn-SOD: cytoplasmic cupper/zinc-superoxide dismutase
DAF-2A: dye 4,5-diaminofluorescein diacetate
DETCA: diethyldithiocarbamate
ECL: electrochemiluminescence
EC-SOD: extracellular cupper/zinc-superoxide dismutase
E_max_: maximum vasodilator effect
eNOS: endothelial nitric oxide synthase
FH: familial hypercholesterolemia
H_2_O_2_: hydrogen peroxide
KCl: potassium chloride
LDL: low-density lipoprotein
LDLr^−/−^: LDL receptor-deficient mice
LDLr^−/−^Ex: exercised LDL receptor-deficient mice group
LDLr^−/−^S: sedentary LDL receptor-deficient mice group
LDLr: LDL receptor
LN: L-NAME
L-NAME: N-nitro-l-arginine methyl ester
Mn-SOD: mitochondrial manganese-superoxide dismutase
MnTMPyP: Mn(III) tetrakis (1-methyl-4-pyridyl) porphyrin pentachloride
NADPH oxidase: nicotinamide-adenine dinucleotide phosphate oxidase
nNOS: neuronal nitric oxide synthase
NO: nitric oxide
NOS: nitric oxide synthase
Nox1: subunit of NADPH oxidase 1
Nox2: subunit of NADPH oxidase 2
Nox4: subunit of NADPH oxidase 4
O_2_: oxygen
p-eNOS: serine 1177-phosphorylated eNOS
PVDF: polyvinylidene fluoride
ROS: reactive oxygen species
SEM: standard error mean
SOD: superoxide dismutase
TG: triglyceride
WT Ex: exercised wild-type group
WT S: sedentary wild-type group
WT: wild-type mice
